# The Dyad Dilemma: Strategies to Recruit Study Partners for Mild Cognitive Impairment Clinical Trials

**DOI:** 10.1093/geroni/igab046.123

**Published:** 2021-12-17

**Authors:** Allison Gibson, Caitlin Pope, Elizabeth Rhodus, Kelly Parsons, Julia Johnson, Dawn Oaks, Greg Jicha

**Affiliations:** 1 University of Kentucky, University of Kentucky, Kentucky, United States; 2 University of Kentucky, Lexington, Kentucky, United States; 3 University Of Kentucky, Sanders-Brown Center On Aging, Lexington, Kentucky, United States

## Abstract

Mild cognitive impairment (MCI) research faces challenges to successful enrollment, especially with clinical trial studies. This study explores researchers’ experiences recruiting from a U.S. Alzheimer’s Disease Center for a pilot, platform trial of biopsychosocial interventions for MCI dyads. Individuals with MCI that met the inclusion criteria for the study were invited to participate (n=39). Thematic analysis of recruitment case notes was utilized to track participants’ and study partners’ interest in participation. In most cases, participants with MCI were interested and willing to enroll and study partners were not. Recruiting persons with MCI and their study partners for clinical trials research may require specialized communication messaging such as education about how interventions address the needs of MCI, along with training on the relationship of MCI to cognitive decline. This presentation highlights effective strategies to engage study partners into recruitment for MCI research such as creating more flexible participation roles and offerings.

